# The effect of occlusal restoration and loading on the development of abfraction lesions: A finite element study

**DOI:** 10.4103/0972-0707.45250

**Published:** 2008

**Authors:** Gaurav Vasudeva, Poonam Bogra

**Affiliations:** Department of Conservative Dentistry and Endodontics, Gian Sagar Dental College and Hospital, Banur, Dist. Patiala, Punjab, India; 1 Department of Conservative Dentistry and Endodontics, DAV Dental College and Hospital, Yamunanagar, Haryana, India

**Keywords:** Abfraction lesion, finite element analysis, occlusal restoration

## Abstract

**Background:**

Abfraction, a type of non-carious cervical tooth loss, is a poorly understood condition. One factor thought to contribute to the development of these lesions is the effect of occlusal loading and the presence of occlusal restoration.

**Aim and Objectives:**

The aim of this paper is to study the stress profile in the cervical region of mandibular first premolar with variation of occlusal loads, and to compare the stress profile between sound and occlusally restored tooth under variation of occlusal load, using two-dimensional plane strain finite element model.

**Materials and Methods:**

A mandibular first premolar was sectioned and modeled in the finite element software, along with its peridontium. Varying occlusal loads were applied along the cuspal inclines, with and without an occlusal restoration. The software used was NISA II EMRC.

**Result:**

It was found that higher occlusal loads caused more cuspal flexure and that the maximum shear stress was much higher and closer to the cervical area. It was also observed that there was a slight increase in shear stress when occlusal restoration was present.

**Conclusion:**

It was suggested that high occlusal loading and the presence of an occlusal amalgam restoration increased the stress concentration at the cervical area, which may lead to the breakdown of enamel at the cervical region.

## INTRODUCTION

For many years, practicing dentists have accepted the theory that hard-bristled toothbrushes coupled with improper brushing technique cause non-carious cervical lesions.[1] Some authors did not believe that the brush could produce the kind of wasting to the hard tissue of the teeth, as was described. It was suggested that brushes might be responsible for injury to the teeth near the gum margin, but in cases where a brush had not been used at all, the injury could not have been caused by severe brushing.[2] Some also found that there was no relationship between cervical abrasion and tooth brushing technique, frequency of brushing the teeth, brand of dentifrice, brand of toothbrush, and/or salivary pH.[3,4] In addition, there was no relationship between the prevalence of cervical abrasion and race or hand dexterity.[5] From data compiled, based on the observation of hundreds of historical human skulls, a hypothesis was raised that these lesions maybe caused by occlusal loading in centric and eccentric movements that may flex the cusp.

Certain experiments were done using engineering principles and forces applied within a tooth were studied when external loads were placed on it using the “Finite Mathematical Element Stress Analysis”; the stress load in teeth were determined. The amount of load placed on the teeth was the key factor.[6] It was found that vertical forces were less harmful because they provided axial stimulation to the teeth and bone. Horizontal forces, however, were extremely damaging, because they subjected the teeth and the bone to torquing and off-loading.[7] It was found that lateral forces and occlusal forces, which cause cuspal flexure, are the cause of the breakdown of tooth structure in the cervical region. It was proposed that bruxing produced most of the destructive forces on the tooth structure.[8] Therefore, Grippo coined the term abfraction. He stated that abfractions were the pathologic loss of both enamel and dentin caused by biomechanical loading forces that lead to flexure and ultimate material fatigue of susceptible teeth at locations away from the point of loading. The forces could be static, as in swallowing and clenching, or cyclic, as in chewing. The breakdown was dependent on the magnitude, duration, direction, frequency, and location of the forces.[9]

Various biomechanical studies have demonstrated the weakening effect of cavity preparations on teeth. It was observed through experiments that progressive removal of tooth substance during cavity preparation led to increased cuspal flexure under occlusal loads.[10] Thus the aim of this study was to investigate the stress profile in the cervical region of a mandibular first premolar, obtained with variation of occlusal loads, and to compare a sound and an occlusally restored tooth, using a two-dimensional finite element analysis.

### Aims and Objectives

1. To study the stress profile and the effect of variation of occlusal load in the cervical region of mandibular first premolar.
2. To compare the stress profile between sound and occlusally restored tooth under variation of occlusal load, using two-dimensional plane strain finite element model.

## MATERIALS AND METHODS

An extracted mandibular first premolar was sectioned buccal-lingually through the center of the tooth and ground to a very thin section, such that the enamel, dentin, cemental and the Pulpal outline was clearly visible.

The exact dimensions were determined and traced on a tracing paper, including the outline of dentino-enamel junction and the pulp. The image was then scanned and modeled in the AutoCAD 2000 software. Once the model was completely prepared, it was captured by the Finite Element Software (NISA II, EMRC).

The outline of the periodontal ligament 0.2 mm wide and the surrounding alveolar bone was generated using the outline of the tooth as a guide. The dimensions of the compact and cancellous bone forming the alveolus were derived from the standard text.[11] The pulp was modeled as a void, since this has been shown to have no effect on the results.[12]

A two-dimensional finite element plane strain mesh of the lower first premolar and supporting ligament and bone was developed from the original geometry, using NISA II FINITE ELEMENT SUITE (EMRC) containing 7774 nodes and 3923 hexahedron elements.

The enamel was modeled as an anisotropic material, with the principal elastic modulus Ex = 84Gpa and Ey = Ez = 20Gpa.[13] The physical properties of the tooth structure and the dental materials used in this analysis were incorporated by published text.[13,14]

An initial pilot study was done and a load 0.5 mm inside the buccal cusp tip produced great stress around the cervical region.

The first variable investigated was with a 100N load 0.5 mm, inside the buccal cusp tip. The load was at right angle to the tooth, as in the case of the eccentric occlusal contacts that lead to lateral forces. Further, the load was increased to 500N. The maximum shear stresses in the buccal enamel in the cervical region were sampled along two horizontal planes. The first plane A-A′ was 1.5 mm above the CEJ and B-B′ 0.5 mm above CEJ.

The maximum shear stresses were sampled at four nodal points in relation to A-A′ plane and three nodal points in relation to B-B′ plane. The average of these nodal points was calculated as the maximum shear stresses in that particular plane. The second variable investigated was in the presence of an occlusal silver amalgam restoration, which was modeled in the finite element profile prepared. The cavity depth was held 0.5 mm inside the DEJ and the width was ¼ of the intercuspal distance. As amalgam forms no bond with enamel and dentin, it was assumed that there was a discontinuity around the occlusal cavity wall interface. This was modeled using specialized elements called gap elements, which are line elements joining two nodes. The layer of the gap element was 1 micrometer wide, which was used to model the interface between the occlusal amalgam restoration and the tooth. Again, 100N load was applied and the maximum shear stresses were calculated at the plane A-A′ and B-B′. The load was then increased to 500N and the maximum shear stresses were calculated at the same planes.

## RESULTS

The results of the maximum shear stresses found across the plane A-A′ and B-B′ were recorded and given in Table 1.

**Table 1:** Mean maximum shear stresses found across planes A-A′ and B-B′

Occlusal restoration	Load	A-A′ plane (Mpa)	B-B′ plane (Mpa)
No occlusal restoration	100 N	22.25	39.00
	500 N	111.50	186.65
Silver amalgam	100 N	23.65	40.19
Restoration	500 N	123.50	193.0

[Figures 1A and B] show the stress pattern during the finite element analysis, when the occlusal load was 100N and 500N respectively.

**Figure 1A:**
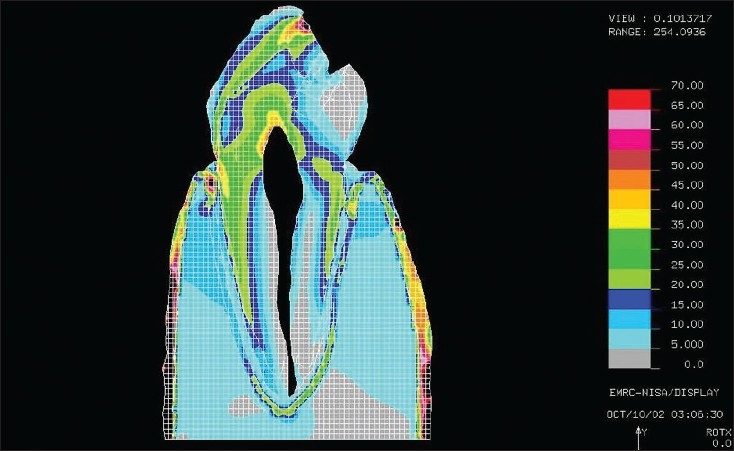
The maximum shear stresses found around the cervical area under a 100N load

**Figure 1B:**
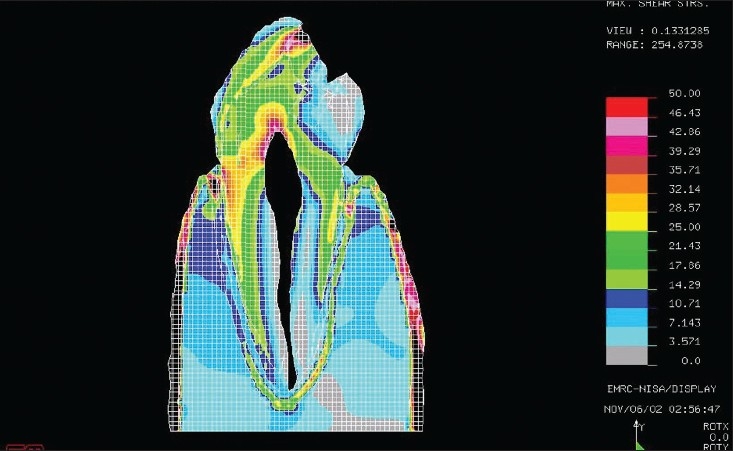
The maximum shear stresses found around the cervical area under a 500N load

Table 2 shows the mean shear stresses, standard deviation and error mean on plane A-A′ and B-B′ under different loads, with and without the presence of an occlusal silver amalgam restoration.

**Table 2:** Maximum shear stresses across planes A-A′ and B-B′

	Mean	Std. deviation	Std. error mean	t	DF	Significance (≤ 0.050)
Pair A-A′-B-B′	4.485	32.22962	6.11481	-2.761	3	0.070 (Insignificant)

Statistical analysis using a paired t-test was done to compare the maximum shear stresses across plane A-A′ and B-B′ [Table 3] and was found to be statistically insignificant (*P* > 0.050).

**Table 3:** Statistical analysis of the maximum shear stresses seen between planes A-A′ and B-B′

	Mean	Standard deviation	Standard error mean
Plane A-A′	70.2250	54.81083	27.40542
Plane B-B′	114.7100	86.77542	43.38771

## DISCUSSION

The major causes of the increase in malocclusions and abfractions in present modern cultures involve the use of bottle-feeding, pacifiers, and excessive noxious infant habits such as thumb and finger sucking, mental tension and stress, which lead to bruxism. Toothbrushes cannot get much softer than they already are; yet people continue to have notches, even with instructions to lighten the forces and to brush in a proper manner.

The overall study showed that the maximum shear stresses on occlusal loading in the cervical region along both planes A-A′ and B-B′ were very high, which supports the cuspal flexure theory given by Hood,[15,16] who studied the biomechanics of class V cavity and found that there is an increased stress concentration at the buccal cervical with a lateral occlusal load. Lee and Eakle[8] said that there was disruption of bond between the enamel rods, due to shear stresses, which was also suggested by McCoy.[7] Small molecules enter between hydroxyapatite crystals and prevent reestablishment of bonds, to make the crystals more susceptible to breakage and chemical dissolution. They also said that as the occlusal load was increased to 500N, there were extremely high shear stresses seen in the cervical area. These results comply with the previous studies which suggest the association of abfraction lesions with higher occlusal loading and lateral loading during eccentric jaw movements, like in bruxism Xonga,[17] Lambrechts.[18] It was also seen that the shear stresses produced at plane B-B′ were much more, as compared to plane A-A′. The reason can be explained on the basis of simple mechanics - if we considered the tooth and its supporting tissues as a biomechanical unit, a simple class 3 lever case applies. The occlusal loads applied near the cusp tips were far away as possible from the fulcrum point of the “beam” if we considered the tooth as a cantilever beam. The fulcrum is between the cervical region of the tooth and the crest of the alveolar bone. Thus, there is an increased cuspal flexure seen in these cases. The points closer to the fulcrum undergo greater stress, as compared to the ones away from it. Though the results were statistically insignificant [Table 3], which could be due to the very small sample size, the stresses produced have shown to exceed the reported failure stresses for enamel given in various studies.[19–22]

It was also seen that in the presence of an occlusal amalgam restoration, the cuspal flexure increased and the maximum shear stresses at the cervical region also increased, but only to a small extent, as observed before.[14,19,20] The reason for this could be the ideal, small size of the amalgam restoration taken in this study; whereas, in usual clinical situations, the occlusal cavities are usually much larger or may be involving the proximal surface that may lead to much higher stress at the cervical area. Previous studies by Vale[23] and later by Hood[10,16] used a steel ball over the occlusal surface to induce load on the tooth structure and through a strain gauge technique it was seen that as the size of occlusal restoration gets larger, the tooth structure weakens and more stresses are seen on the tooth.

However, the results of this study must be viewed with a few limitations in mind, since this study is only a two-dimensional analysis, whereas teeth are three-dimensional objects. Therefore, this model was unable to model the torsional movements that may occur in the mesial-distal plane on occlusal loading, which could lead to an underestimation of the cervical stresses.

Future scope lies with the three-dimensional analysis with various types of cavities modeled. It can also be suggested that if the occlusal restoration were composite instead of silver amalgam, the cuspal flexure would further reduce due to the bonding ability of composites and the modulus of elasticity being much closer to the tooth structure.[19,20] As only an ideal occlusal class I restoration was modeled, the effect of a class II or a MOD would only be more detrimental to cervical enamel and will be interesting to observe.

## CONCLUSION

The study demonstrated that stresses closer to cementoenamel junction were much more than those that were away from it. High lateral occlusal loading leads to increased stress concentration at the cervical region, due to increased cuspal flexure. The amount of shear stresses produced in this study was more than the amount of stress suggested by previous studies that led to breakdown of enamel in that area. It was also seen that the presence of occlusal amalgam restoration increased the stresses at the cervical region, as compared to a sound tooth.
